# Implementation of High Time Delay Accuracy of Ultrasonic Phased Array Based on Interpolation CIC Filter

**DOI:** 10.3390/s17102322

**Published:** 2017-10-12

**Authors:** Peilu Liu, Xinghua Li, Haopeng Li, Zhikun Su, Hongxu Zhang

**Affiliations:** State Key Laboratory of Precision Measuring Technology and Instruments, Tianjin University, Tianjin 300072, China; liupeilu@tju.edu.cn (P.L.); lhp19911119@126.com (H.L.); suzhikun@tju.edu.cn (Z.S.); zhanghongxv123@tju.edu.cn (H.Z.)

**Keywords:** ultrasonic phased array, time delay accuracy, interpolation, CIC filter, parallel decomposition, compensation, FPGA

## Abstract

In order to improve the accuracy of ultrasonic phased array focusing time delay, analyzing the original interpolation Cascade-Integrator-Comb (CIC) filter, an 8× interpolation CIC filter parallel algorithm was proposed, so that interpolation and multichannel decomposition can simultaneously process. Moreover, we summarized the general formula of arbitrary multiple interpolation CIC filter parallel algorithm and established an ultrasonic phased array focusing time delay system based on 8× interpolation CIC filter parallel algorithm. Improving the algorithmic structure, 12.5% of addition and 29.2% of multiplication was reduced, meanwhile the speed of computation is still very fast. Considering the existing problems of the CIC filter, we compensated the CIC filter; the compensated CIC filter’s pass band is flatter, the transition band becomes steep, and the stop band attenuation increases. Finally, we verified the feasibility of this algorithm on Field Programming Gate Array (FPGA). In the case of system clock is 125 MHz, after 8× interpolation filtering and decomposition, time delay accuracy of the defect echo becomes 1 ns. Simulation and experimental results both show that the algorithm we proposed has strong feasibility. Because of the fast calculation, small computational amount and high resolution, this algorithm is especially suitable for applications with high time delay accuracy and fast detection.

## 1. Introduction

Ultrasonic phased array detection technology is a kind of technology combining phased array theory with traditional ultrasonic detection [1,2]. By performing phase delay control on each element in ultrasonic array transducer, beamforming and phased focusing are realized, and the non-destructive test can be performed on the workpiece with complex geometric shape [3]. The core of ultrasonic phased array detection technology is to achieve the launch of ultrasonic and deflection or focusing of echo signal by controlling the phase of transducer excitation signal and echo signal precisely. Common time delay methods are analog line time delay, delay chip, sampling time delay, digital time delay [4,5,6]. The analog line time delay requires a lot of LC network and electronic switch matrix, with low time delay accuracy, low integration and poor anti-interference. Sampling time delay and delay chip can achieve high time delay accuracy, but with high cost, poor portability and low flexibility.

Because of the high precision, good stability, flexible control, versatility, high portability, digital time delay has become the focus of research in recent years [7,8,9]. By calculating the phase difference, Wang Junlin achieved high-intensity phased array focus [10]. Cruza used dynamic focusing technology to achieve the precise focus of ultrasonic phased array [11]. By improving the 8× interpolation structure, Liu Guixiong used multi-stage half-band filter to achieve 1.25 ns high-precision delay [12]. Md Omar Khyam proposed a highly accurate time-of-flight measurement technique based on phase-correlation for ultrasonic ranging [13]. Although the above methods can achieve high time delay accuracy, the algorithms are more complicated and more difficult to achieve in hardware implementations. In addition, using Phase-Locked Loop (PLL) multiplication and phase shifting of FPGA, high time delay accuracy can also be achieved [14], but the clock must work at a higher frequency, so the selection of FPGA becomes smaller and timing constraints become more difficult.

CIC filter has been widely used in hardware implementations because of its simple structure, fast operation speed and small resource occupation [15,16,17]. Based on FPGA, we 8× interpolated the CIC filter and decomposed the echo signal to eight channels. In the case of system clock is 125 MHz, high time delay accuracy of 1 ns is realized. We also summarized the general formula of arbitrary multiple interpolation CIC filter parallel algorithm. Moreover, we improved the structure of the 8× interpolation CIC filter parallel algorithm, so that the speed of computation can be insured while computational amount can be reduced. Faced with the CIC filter’s pass band is not flat, transition band is not steep and narrow, and stop band suppression is not strong enough [18], we compensated it. In order to verify the feasibility of the algorithm, we performed simulation on Modelsim and carried out a defect echo detection experiment. Simulation and experimental results both show that the algorithm we proposed has high feasibility in hardware implementations, and can be used to achieve high time delay accuracy of 1 ns successfully. Finally, in order to verify the effect of time delay accuracy on defect detection, we did a comparative test, using the same set of equipment with different time delay accuracies to test the same defects. Experimental results show that compared with 2 ns time delay accuracy, the result of defect detection with 1 ns time delay accuracy is more accurate.

## 2. Interpolation CIC Filter Parallel Algorithm

### 2.1. CIC Filter with Traditional Structure

The CIC filter with a traditional structure has no multipliers, only adders, integrators and registers, is highly suitable for high sampling rate conditions, so we can achieve digital upconversion by interpolating it [15].

In this paper, the phased array system clock is 125 MHz. In order to achieve 1 ns time delay accuracy on FPGA, we need to use the PLL multiply 50 MHz (crystal) clock to 125 MHz, then 8× interpolate sampling rate from 125 MHz to 1000 MHz.

**Figure 1:**
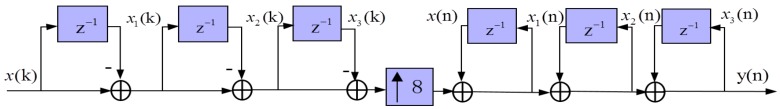
8× interpolation CIC filter with three stage cascade structure.

If we 8× interpolate the traditional three stage cascade CIC filter, as shown in Figure 1, even though it only has adders without multipliers and operates efficiently, the data sampling rate of input sequence x(k) (at a sampling rate of 125 MHz) becomes 1000 MHz after eight times multiplication. Considering the existing resources of FPGA, it is very difficult to achieve the adder at a processing speed of 1000 MHz, which makes it impossible to achieve the algorithm of 1 ns time delay accuracy in hardware implementations.

### 2.2. Interpolation CIC Filter Parallel Algorithm

From Figure 1, we obtain the following eight relations:

(1) $$x_{1}(k)=x(k)-x(k-1)$$

(2) $$x_{2}(k)=x_{1}(k)-x_{1}(k-1)$$

(3) $$x_{3}(k)=x_{2}(k)-x_{2}(k-1)$$

(4) $$x(n)=\{\begin{array}{l} x_{3}(k),\;n=8k;\;k=0,1,\cdot \cdot \cdot ,N-1\\ 0,\;n=8k+1,8k+2,\cdot \cdot \cdot ,8k+7\; \end{array}$$

(5) $$x_{1}(n)=x(n)+x_{1}(n-1)$$

(6) $$x_{2}(n)=x_{1}(n)+x_{2}(n-1)$$

(7) $$x_{3}(n)=x_{2}(n)+x_{3}(n-1)$$

(8) $$y(n)=x_{3}(n)$$

where *N* is the length of input sequence x(k).

Assume input sequence $x(k)=\{0,m_{1},m_{2},m_{3},m_{4},m_{5},m_{6},m_{7},...\}$, from (1)–(3), we obtain:

$$\begin{array}{cc} x_{3}(k)= & \{0,m_{1},m_{2}-3m_{1},m_{3}-3m_{2}+3m_{1},m_{4}-3m_{3}+3m_{2}-m_{1},m_{5}-3m_{4}\\ & \;+3m_{3}-m_{2},m_{6}-3m_{5}+3m_{4}-m_{3},m_{7}-3m_{6}+3m_{5}-m_{4},...\} \end{array}$$

According to (4), we 8× interpolate $x_{3}(k)$, inserting seven 0 into every adjacent sequence of $x_{3}(k)$, from (5)–(8), then we obtain the output sequence:

$$\begin{array}{cc} y(n)= & \{0,...,0,m_{1},3m_{1},6m_{1},10m_{1},15m_{1},21m_{1},28m_{1},36m_{1},m_{2}+42m_{1},3m_{2}+46m_{1},\\ & \;6m_{2}+48m_{1},10m_{2}+48m_{1},15m_{2}+46m_{1},21m_{2}+42m_{1},...,36m_{6}+28m_{6},...\} \end{array}$$

Decomposing y(n) to 8 sequence $y_{0}(n)$, $y_{1}(n)$, $y_{2}(n)$, $y_{3}(n)$, $y_{4}(n)$, $y_{5}(n)$, $y_{6}(n)$, $y_{7}(n)$, in order to observe them conveniently, we summarize the formula of 8× interpolation CIC filter parallel algorithm:

(9) $$y(n)=\{\begin{array}{ll} x(k)+42x(k-1)+21x(k-2), & n=8k\\ 3x(k)+46x(k-1)+15x(k-2), & n=8k+1\\ 6x(k)+48x(k-1)+10x(k-2), & n=8k+2\\ 10x(k)+48x(k-1)+6x(k-2), & n=8k+3\\ 15x(k)+46x(k-1)+3x(k-2), & n=8k+4\\ 21x(k)+42x(k-1)+x(k-2), & n=8k+5\\ 28x(k)+36x(k-1), & n=8k+6\\ 36x(k)+28x(k-1), & n=8k+7 \end{array}$$

Similarly, we can obtain the formulas of 4×, 5×, 6×, 7×, 9×, 10× interpolation CIC filter algorithm; they are shown in Table 1 for observation and comparison.

Observing and analyzing Table 1, we summarize the general formula of arbitrary multiple interpolation CIC filter parallel algorithm:

(10) $$t_{a,1}=\{\begin{array}{ll} 1, & a=1\\ 2t_{a-1,1}-t_{a-2,1}+1, & 1<a\leq I \end{array}$$

(11) $$t_{a,2}=\{\begin{array}{ll} t_{a-1,1}, & a=I\\ t_{a+1,1}, & a=I-1\\ 2t_{a+1,2}-t_{a+2,2}-2, & (I-1)/2\leq a<I-1\;\\ t_{I-a-1,2}, & 1\leq a<\;(I-1)/2 \end{array}$$

(12) $$t_{a,3}=\{\begin{array}{ll} 0, & a=I\;or\;a=I-1\\ t_{I-a-1,1}, & 1\leq a<\;I-1 \end{array}$$

where *I* is interpolation multiple, *a* is row.

### 2.3. Structure Optimization of 8× Interpolation CIC Filter Parallel Algorithm

Observing (9), we find out the x(k−1) coefficients of $y_{0}(n)$ and $y_{5}(n)$, $y_{1}(n)$ and $y_{4}(n)$, $y_{2}(n)$ and $y_{3}(n)$ are the same. In addition, the coefficients of 0 in the formula can be directly removed and every coefficient of 1 means one multiplier can be reduced. Based on these principles, we simplified the structure of 8× interpolation CIC filter parallel algorithm.

Upon further analysis, assuming the input sequence is x(k), the algorithm takes 24k multiplications and 16k additions before the simplification, while it only takes 17k multiplications and 14k additions after the simplification, eliminating the unnecessary computing steps, reducing 12.5% of addition and 29.2% of multiplication as a result. It goes without saying that the simplification ensures the speed of calculation while maximizing the savings of FPGA area resources and Digital Signal Process (DSP) resources.

**Figure 2:**
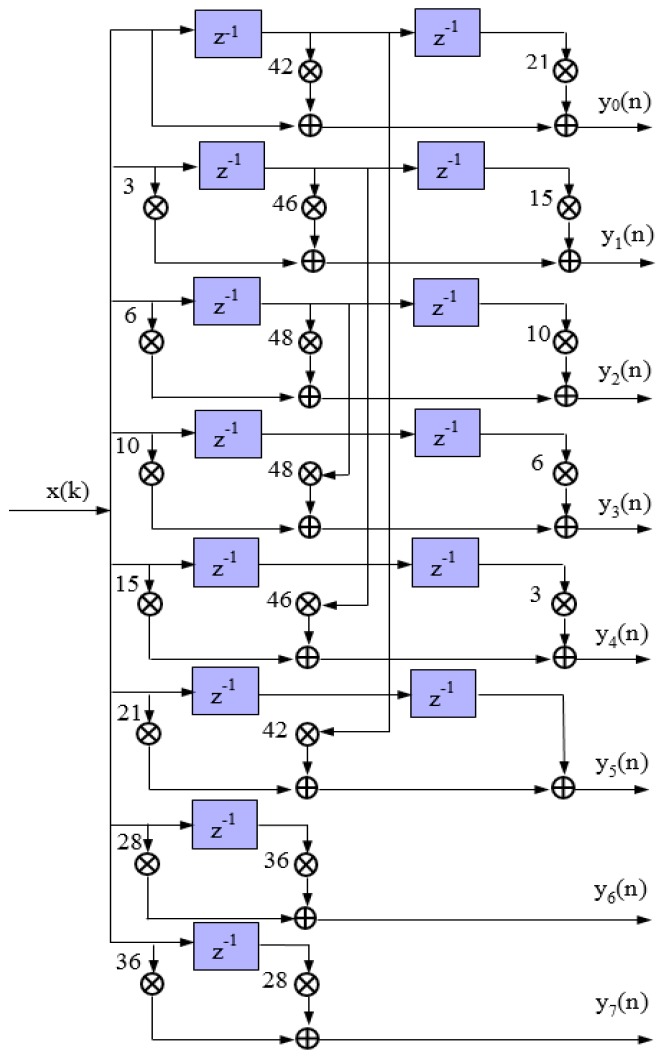
The structure of simplified 8× interpolation CIC filter parallel algorithm.

The structure of simplified 8× interpolation CIC filter parallel algorithm is shown in Figure 2. According to the principle of area for speed in FPGA design, although the use of multipliers makes the structure seem complex, the method of 8-channel data parallel processing allows multipliers and adders to operate at 1/8 original rate, which effectively improves the speed of the interpolation filter and solves the problem that adders cannot operate directly at a rate of 1000 MHz.

**Figure 3:**
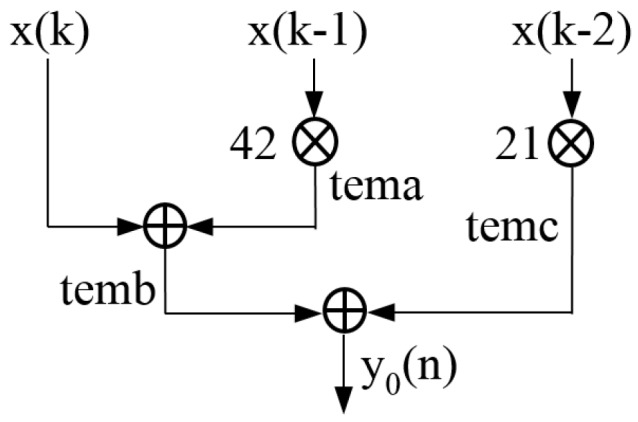
The first channel structure.

In order to further analyze and optimize the structure of the parallel algorithm, we extract the first channel signal, as shown in Figure 3.

The sequence $x_{1}(k)$ is multiplied by coefficient 42 and the multiplier is delayed one clock cycle to obtain the result of the multiplication (we call the result of multiplication tema). If we use tema to add with x(k) directly, there is no doubt that the data will be misaligned, because x(k) is still in the first clock cycle while tema is already in the second clock cycle. So, it is necessary to delay x(k) for one clock cycle and then add it with tema, obtaining the result of addition (we call the result of addition as temb). Similarly, if we use temb to add to the multiplication result of $x_{2}(k)$ and coefficient (we call it temc), the data will also be misaligned. So, we need to delay temc for one clock cycle to the third clock cycle and then add it to temb, eventually obtaining $y_{0}(n)$.

**Figure 4:**
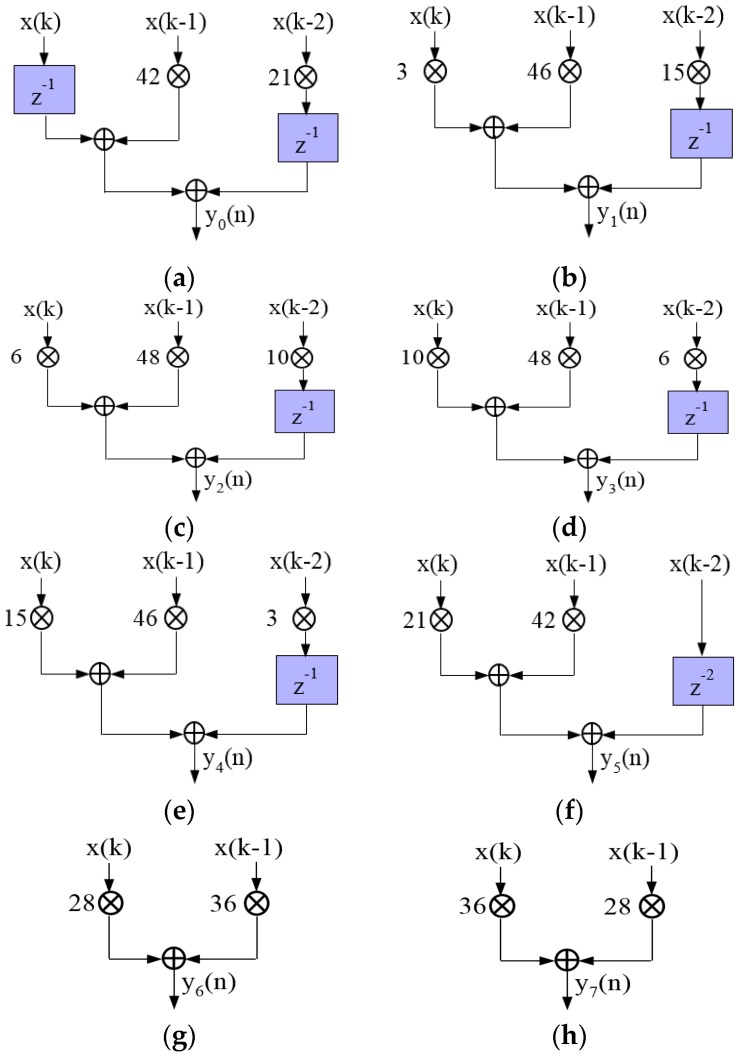
Optimized 8-channel parallel structure: (**a**) $y_{1}(n)$; (**b**) $y_{2}(n)$; (**c**) $y_{3}(n)$; (**d**) $y_{3}(n)$; (**e**) $y_{4}(n)$; (**f**) $y_{5}(n)$; (**g**) $y_{6}(n)$; (**h**) $y_{7}(n)$.

Similarly, we optimize the other seven channel structures, as shown in Figure 4.

### 2.4. The Principle of Ultrasonic Phased Array Focusing Time Delay Based on 8× Interpolation CIC Filter Parallel Algorithm

**Figure 5:**
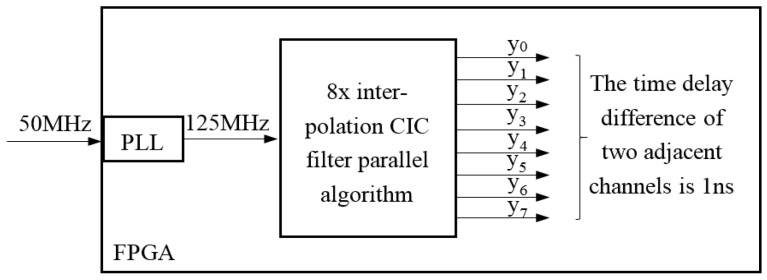
The ultrasonic phased array focusing time delay system based on 8× interpolation CIC filter parallel algorithm.

Based on 8× interpolation CIC filter parallel algorithm, we establish the ultrasonic phased array focusing time delay system as shown in Figure 5.

In this paper, crystal clock is 50 MHz, and after the frequency multiplication of PLL, the clock of the phased array system becomes 125 MHz and the clock cycle is 8 ns. Then after 8× interpolation CIC filter parallel algorithm, the time delay difference of two adjacent channels in 8-channel signals at 125 MHz sampling rate is 1 ns.

## 3. Compensation of CIC Filter

### 3.1. Performance Analysis of CIC Filter

Frequency magnitude response of CIC filter can be expressed as [15]:

(13) $$\left|H(e^{j\omega })\right|={\left|I\frac{\sin(\omega I/2)}{\sin(\omega I)}\right|}^{N}$$

where *I* is the interpolation factor, and *N* is the number of stages.

The disadvantages of the CIC filter are as follows. First, it does not have a flat and wide pass band, which is undesirable in many applications. Second, CIC filter does not offer narrow transition bandwidth and good stop band attenuation alone.

**Figure 6:**
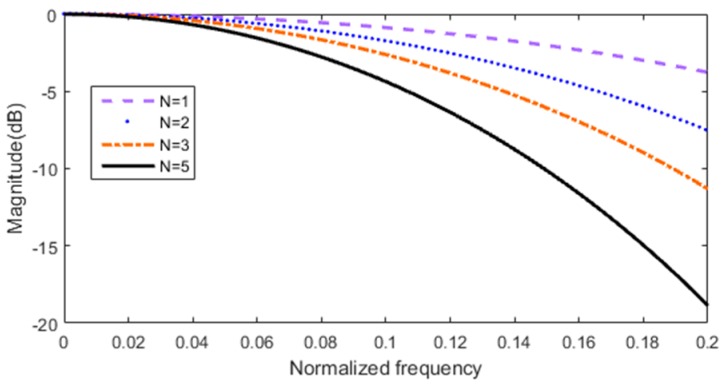
Magnitude response of the 5-order CIC filter with different stages.

Increasing *N* can improve stop band attenuation, but pass band droop will be greater and pass band will accordingly be more uneven. Figure 6 shows magnitude response of the 5-order CIC filter with different stages.

It can be seen from Figure 6, in the case of the same order (5-order), that the higher the stages of CIC filter is, the more obvious the pass band droop is. Therefore, *N* should be decided by the actual situation, generally *N* is less than 5. In this paper, the number of stages *N* = 3.

### 3.2. Compensated CIC Filter

The parameters of 8× interpolation CIC filter are designed as follows, the differential delay factor *D* = 1, the number of stages *N* = 3, and the interpolation factor *I* = 8. As [19] pointed out that when the order of CIC filter M and interpolation factor *I* are equal, we can put the M data into a group, adding them directly to get one required output result, without reusing or discarding some data. In this way, we can achieve CIC filtering and the interpolation process at the same time, so as to achieve the purposes of reducing computational amount and saving hardware resources. So in this paper, *M* = *I* = 8.

There is no doubt that we have to compensate for the CIC filter if we want to use it in the ultrasonic phased array system. In recent years, many researchers have dedicated large efforts to improving frequency magnitude response characteristics by using compensation filters [20,21,22,23,24]. However, most of the compensations are based on decimation CIC filter instead of interpolation CIC filter.

**Figure 7:**
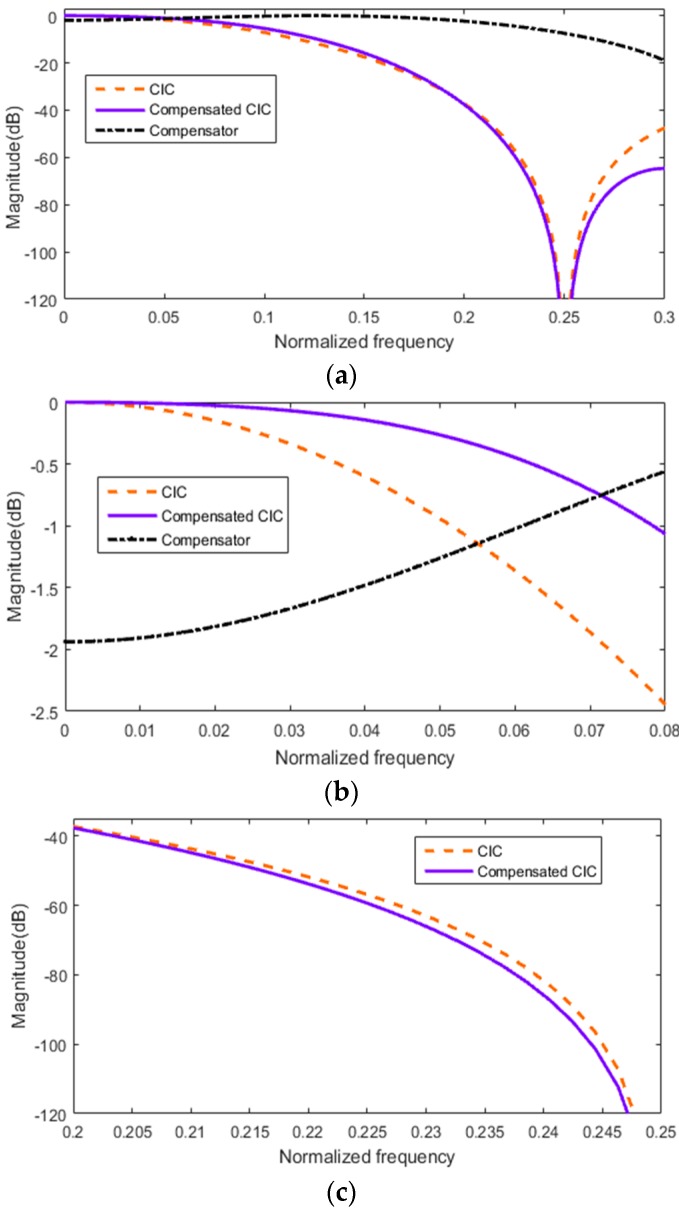
Analysis of CIC compensation. (**a**) Magnitude response of CIC filter before and after compensation; (**b**) pass band characteristics; (**c**) transition band characteristics.

When the value of differential delay factor D is fixed, the frequency magnitude response of CIC filter barely changes with the interpolation factor *I* increasing [16]. When *I* is up to 16, this change can be ignored. Accordingly, it is possible to use a non-recursive Finite Impulse Response (FIR) filter to compensate for droop in the pass band of the CIC filter with different interpolation factors *I*. In addition, taking the lack of a well-defined transition band and stop band attenuation is not fully decreased into consideration; we also need to impose constraints on FIR compensation filter instead of just having wide band compensation. Figure 7 shows magnitude response of the CIC filter before and after compensation.

The original sampling rate of the system is 125 MHz; after 8× interpolation the sampling rate becomes 1000 MHz. The central frequency of the transducer is 3 MHz; its corresponding normalized frequency is 0.056. The frequency of the echo signal is 0.5 MHz–5 MHz, so the corresponding normalized frequency is 0.008–0.08. It can be seen from Figure 7a that the first-order sidelobe attenuation of the CIC filter is only 45 dB before compensation, and it can reach 67 dB after compensation. As we can see from Figure 7b, the maximum attenuation of the pass band is 2.5 dB before compensation, and the maximum attenuation of the pass band is only 1 dB after compensation, so the compensated CIC filter has a low droop and flat pass band. From Figure 7c, we can see that the transition band characteristics have also been improved; the transition band becomes narrower and steeper. Therefore, the compensated interpolation CIC filter can meet the requirements of the phased array system.

## 4. Simulation and Experiments

### 4.1. Simulation of 8× Interpolation CIC Filter Parallel Algorithm

**Figure 8:**
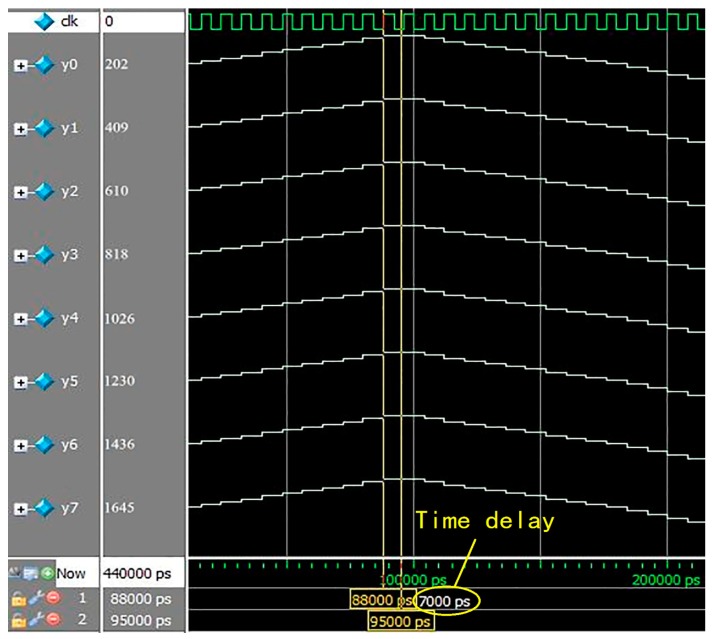
Simulation of 8× interpolation CIC filter parallel algorithm.

In order to verify if the 8× interpolation CIC filter parallel algorithm we proposed is valid, we run the simulation test on the Modelsim; the system clock of FPGA and the sampling rate of echo signal are both 125 MHz. Clk is 125 MHz clock signal, clock cycle is 8 ns. In this simulation, we use the 3 MHz sine signal as the input signal to simulate echo signal, through 8× interpolation filtering and decomposition of the algorithm, the 3 MHz sine signal becomes 8-channel output signals $y_{0}$, $y_{1}$, $y_{2}$, $y_{3}$, $y_{4}$, $y_{5}$, $y_{6}$, $y_{7}$. Figure 8 shows the result of 8× interpolation CIC filter parallel algorithm simulation; we can find that the time delay difference between the first and the eighth output signal is 7 ns, then we can determine that the time delay difference between two adjacent output signals is 1 ns in an indirect way. So, high-precision focusing can be achieved by controlling phased array with 1 ns time delay.

**Figure 9:**
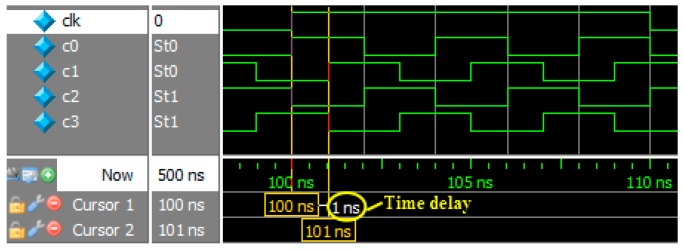
Simulation of PLL multiplication and phase shifting.

As [14] mentioned, high time delay accuracy can be implemented on FPGA by PLL multiplication and phase shifting. However, the clock needs to work at 250 MHz if we want to obtain 1 ns time delay accuracy, so the selection of FPGA becomes smaller and timing constraints become more difficult. Figure 9 shows simulation of PLL multiplication and phase shifting.

Multi-stage half-band filter was used to achieve 1.25 ns high-precision delay [12]. By improving the 8× interpolation structure, the filter is decomposed into eight sub-filters which can simultaneously filter.

The coefficients of the eight sub-filters are:

(14) $$\begin{array}{l} h_{0}(n)=\{0,-21,111,189,-28,1\}\\ h_{1}(n)=\{0,-26,151,151,-26,0\}\\ h_{2}(n)=\{1,-28,189,111,-21,0\}\\ h_{3}(n)=\{2,-25,221,70,-15\}\\ h_{4}(n)=\{2,-17,246,31,-7\}\\ h_{5}(n)=\{0,0,256,0,0\}\\ h_{6}(n)=\{-7,31,246,-17,2\}\\ h_{7}(n)=\{-15,70,221,-22,2\} \end{array}$$

Assuming the input sequence is x(k), we obtain 8-channel output sequences are:

(15) $$\begin{array}{l} y_{0}(n)=-21x(k-1)+111x(k-2)+189x(k-3)-28x(k-4)+x(k-5)\\ y_{1}(n)=-26x(k-1)+151x(k-2)+151x(k-3)-26x(k-4)\\ y_{2}(n)=x(k)-28x(k-1)+189x(k-2)+111x(k-3)-21x(k-4)\\ y_{3}(n)=2x(k)-25x(k-1)+221x(k-2)+70x(k-3)-15x(k-4)\\ y_{4}(n)=2x(k)-17x(k-1)+246x(k-2)+31x(k-3)-7x(k-4)\\ y_{5}(n)=256x(k-2)\\ y_{6}(n)=-7x(k)+31x(k-1)+246x(k-2)-17x(k-3)+2x(k-4)\\ y_{7}(n)=-15x(k)+70x(k-1)+221x(k-2)-22x(k-3)+2x(k-4) \end{array}$$

From Table 2, we know 8× interpolation CIC filter we proposed has obvious advantages over 8× interpolation half-band filter. First, compared to 8× interpolation half-band filter, 8× interpolation CIC filter only uses 17k multiplications, reducing 13k multiplications, which means 18 DSP block 9-bit elements can be saved. Second, 8× interpolation CIC filter only uses 14k additions; this explains why it saves 144 LUTs. Third, 8× interpolation CIC filter does not use any memory bits, however, 8× interpolation half-band filter uses 20 memory bits. In conclusion, both algorithms can implement high time delay accuracy on FPGA, but there is no doubt that the 8× interpolation CIC filter we proposed uses less hardware resources.

**Figure 10:**
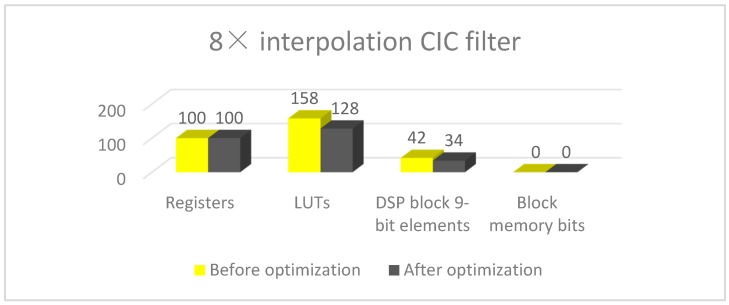
8× interpolation CIC filter hardware costs.

Figure 10 shows 8× interpolation CIC filter hardware costs, 30 LUTs and 8 DSP block 9-bit elements are saved after optimization.

### 4.2. Experiments of Defect Echo Detection

**Figure 11:**
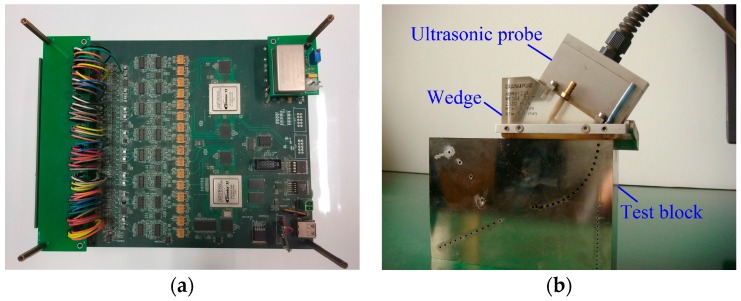
The ultrasonic phased array detection system. (**a**) Circuit board of ultrasonic transmitting and defect echo receiving; (**b**) Ultrasonic probe and standard test block.

**Figure 12:**
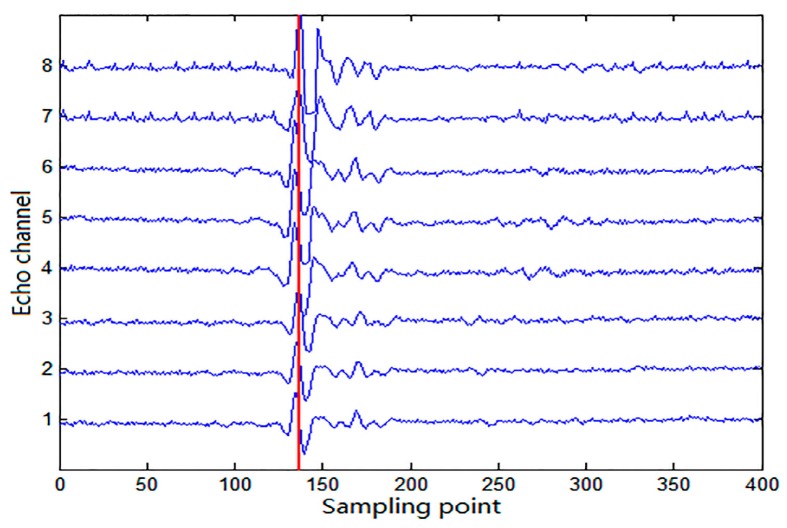
The waveforms of 8-channel defect echo signals.

The ultrasonic phased array defect detection system of this paper is shown in Figure 11. The circuit board contains 64 ultrasonic transmitting channels and 32 echo receiving channels. Wedge is put on test block connected with ultrasonic probe. Central frequency of ultrasonic probe is 3 MHz. The purpose of this experiment is to test if the algorithm we purposed is valid when we use it to interpolation filter and decompose the defect echo. In this experiment, the system clock of FPGA and the sampling rate of echo signal are both 125 MHz; we use single channel to complete ultrasonic transmitting and echo signal receiving. With 8× interpolation filtering and decomposition of the algorithm, the defect echo signal becomes 8-channel signals, as we can see in Figure 12.

Observing and analyzing the waveforms we can find, because the sampling rate of each channel is 125 MHz, every sampling point corresponds to one clock cycle 8 ns. As the red line shows in the waveforms, if we assume the first channel corresponds to 0 ns–1 ns in every 8 ns, similarly, we can also know the eighth channel corresponds to 7 ns–8 ns. Accordingly, we calculate the time delay difference between the first and the eighth channel of the defect echo signal is 7 ns, obtaining the time delay difference between two adjacent signals is 1 ns. Therefore, the 8× interpolation CIC filter parallel algorithm we proposed in this paper has good practicability in defect echo detection experiment.

**Figure 13:**
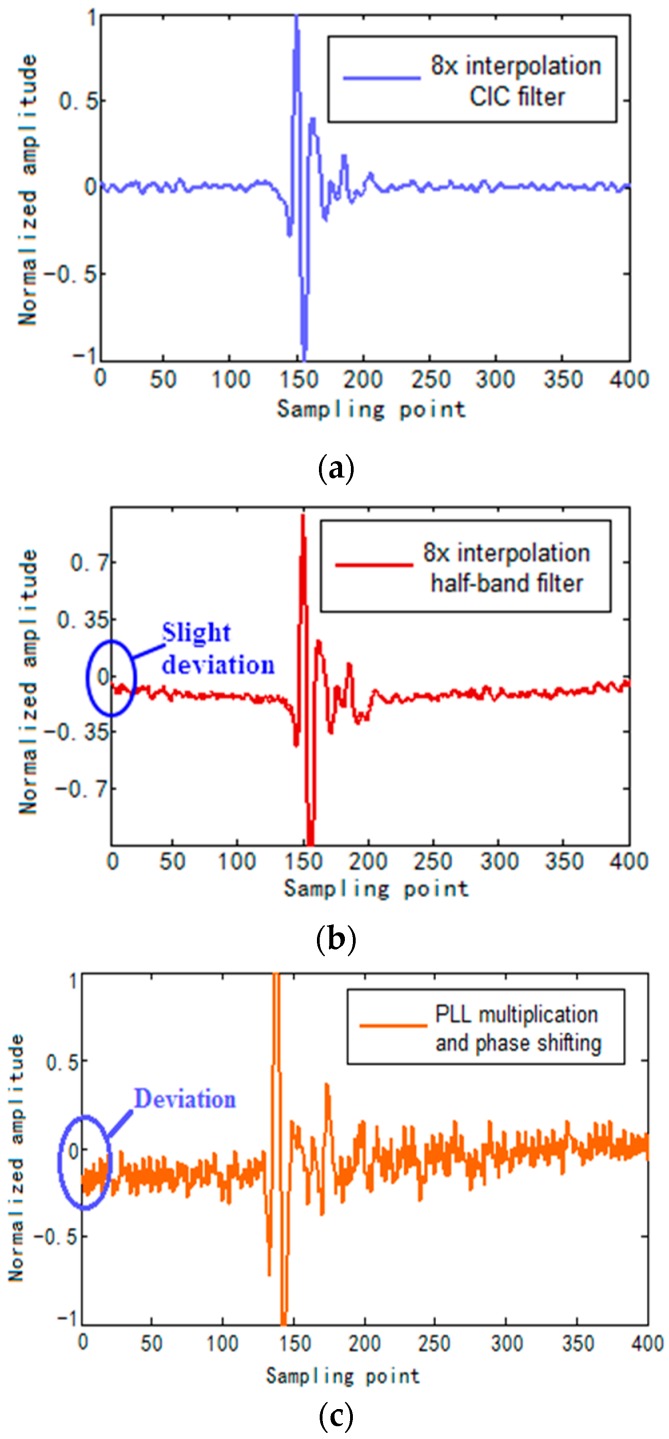
Synthesized A-scan defect echo signal. (**a**) 8× interpolation CIC filter; (**b**) 8× interpolation half-band filter; (**c**) PLL multiplication and phase shifting.

Figure 13 shows synthesized A-scan defect echo signal. As we can see from Figure 13c, the defect echo signal deviates from the horizontal line, clutter and noise is severe. Because the clock works at 250 MHz and timing constraints become more difficult, resulting in the echo signal becoming unsteady. From Figure 13a,b we know, slight deviation exists in the defect echo signal of 8× interpolation half-band filter while there is no deviation existing in the defect echo signal of 8× interpolation CIC filter.

**Figure 14:**
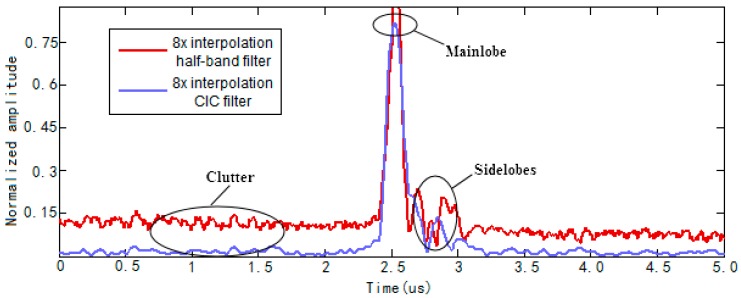
Envelope curves of synthesized A-scan defect echo signals.

In order to further analyze the A-scan defect echo signals of the two algorithms, we extracted envelope curves of synthesized A-scan defect echo signals. As we can see from Figure 14, compared to 8× interpolation half-band filter, envelope curve of 8× interpolation CIC filter has smaller sidelobes and clutter, which means it has higher Signal Noise Ratio (SNR). In addition, the clutter fluctuation of 8× interpolation half-band filter envelope curve is stronger than that of 8× interpolation CIC filter. So the synthesized A-scan defect echo signal of 8× interpolation CIC filter has higher SNR and stronger steadiness.

### 4.3. Experiments of Time Delay Accuracy

In order to further illustrate the effect of time delay accuracy on the defect detection of ultrasonic phased array system, we use the ultrasonic phased array detection system (as shown in Figure 11) to perform defect detection experiments with 2 ns and 1 ns time delay accuracy respectively. The system clock is 125 MHz in all the experiments.

**Figure 15:**
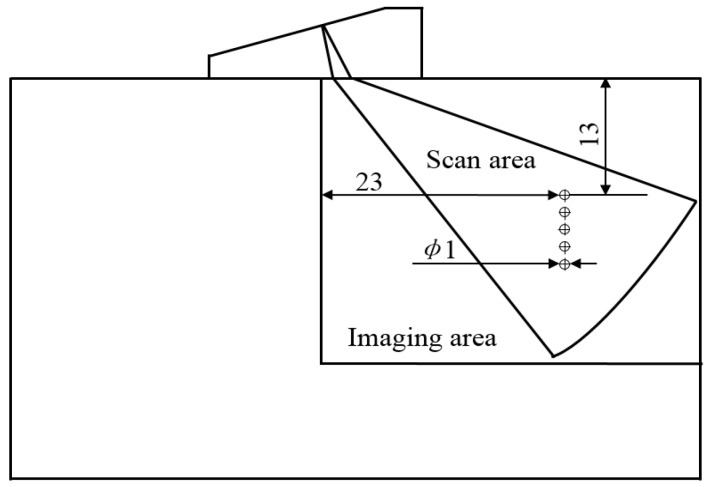
Ultrasonic phased array focusing section scan.

**Figure 16:**
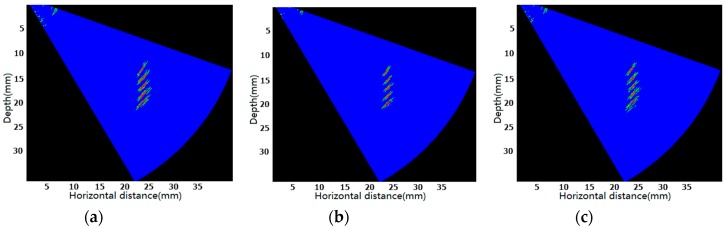
The results of ultrasonic phased array sector scan with different time delay accuracies: (**a**) 2 ns (4× interpolation CIC filter); (**b**) 1 ns (8× interpolation CIC filter); (**c**) 1 ns (8× interpolation half-band filter).

The defects are one column 1 mm through-holes as shown in Figure 15. Figure 16 shows the results of ultrasonic phased array sector scan with different time delay accuracies.

Where red represents the defects, green on behalf of the sidelobes. From Figure 16a,b we know, using the interpolation CIC filter parallel algorithm we proposed in this paper with different interpolation multiple 4× and 8×, that we can obtain 2 ns and 1 ns time delay accuracies respectively. However, Figure 16b has smaller sidelobes and more concentrated energy, which means higher time delay accuracy can image better and locate defect position more accurately. From Figure 16b,c we know, in the case of same time delay accuracy (1 ns) with different filtering algorithms, Figure 16b,c both locate defects position accurately. However, given that Figure 16b has smaller sidelobes, there is no doubt that Figure 16b images better than Figure 16c.

**Figure 17:**
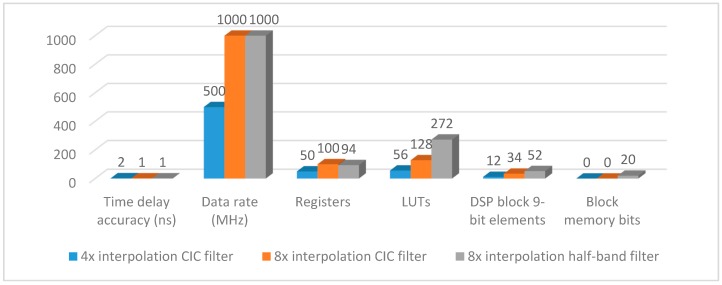
Performances and hardware costs.

It can be seen from Figure 17, compared to 4× interpolation CIC filter, 8× interpolation CIC filter can obtain higher time delay accuracy and faster data processing rate, but with more hardware costs as a result. Based on the general formula of arbitrary multiple interpolation CIC filter parallel algorithm we have proposed in this paper, that readers can choose suitable interpolation multiple to achieve upsampling in different cases.

## 5. Conclusions

In this paper, we proposed an 8× interpolation CIC filter parallel algorithm and achieved this algorithm on FPGA. In the case of system clock is 125 MHz, multichannel decomposing defect echo signal into 8 channels, we obtained 1 ns time delay accuracy signal and solved the problem that adders cannot work at the rate of 1000 MHz directly. Moreover, analyzing the structures of different multiple interpolation CIC filter parallel algorithm, we generalized the interpolation CIC filter algorithm and summarized the general formula of arbitrary multiple interpolation CIC filter parallel algorithm. Optimizing the structure of 8× interpolation CIC filter parallel algorithm, 12.5% of addition and 29.2% of multiplication was reduced. In addition, considering the existing problems of the CIC filter, we compensated CIC filter, the compensated CIC filter’s passband is more flatter, the transition zone becomes steep, and the stopband attenuation increases. Simulation and experimental results both show that the algorithm has high feasibility, fast calculation, small computation and high resolution, which is of great practical significance to improve the performance of the whole ultrasonic phased array instruments.

## Figures and Tables

**Table 1:** Interpolation coefficients of different interpolation multiples.

4×			5×			6×			7×			8×			9×			10×		
1	12	3	1	18	6	1	25	10	1	33	15	1	42	21	1	52	28	1	63	36
3	12	1	3	19	3	3	27	6	3	36	10	3	46	15	3	57	21	3	69	28
6	10	0	6	18	1	6	27	3	6	37	6	6	48	10	6	60	15	6	73	21
10	6	0	10	15	0	10	25	1	10	36	3	10	48	6	10	61	10	10	75	15
			15	10	0	15	21	0	15	33	1	15	46	3	15	60	6	15	75	10
						21	15	0	21	28	0	21	42	1	21	57	3	21	73	6
									28	21	0	28	36	0	28	52	1	28	69	3
												36	28	0	36	45	0	36	63	1
															45	36	0	45	55	0
																		55	45	0

**Table 2:** Performances of the 8× interpolation CIC filter and 8× interpolation half-band filter.

Algorithm	8× Interpolation CIC Filter	8× Interpolation Half-Band Filter
Multiplications	17k	30k
Additions	14k	27k
LUTs	128	272
Registers	100	94
DSP block 9-bit	34	52
Memory bits	0	20
